# Opportunities and risks of self-binding directives: A qualitative study involving stakeholders and researchers in Germany

**DOI:** 10.3389/fpsyt.2022.974132

**Published:** 2022-10-21

**Authors:** Sarah Potthoff, Marleen Finke, Matthé Scholten, Astrid Gieselmann, Jochen Vollmann, Jakov Gather

**Affiliations:** ^1^Institute for Medical Ethics and History of Medicine, Ruhr University Bochum, Bochum, Germany; ^2^Department of Psychiatry, Campus Benjamin Franklin, Charité – Universitätsmedizin Berlin, Berlin, Germany; ^3^Department of Psychiatry, Psychotherapy and Preventive Medicine, LWL University Hospital, Ruhr University Bochum, Bochum, Germany

**Keywords:** psychiatric advance directive, advance statement, Ulysses arrangement, joint crisis plan, mental healthcare, coercion

## Abstract

**Purpose:**

Self-binding directives (SBDs) are a special type of psychiatric advance directive in which mental health service users can consent in advance to involuntary hospital admission and involuntary treatment during future mental health crises. This study presents opportunities and risks of SBDs reported by users with bipolar disorder, family members of people with bipolar disorder, professionals working with people with bipolar disorder and researchers with expertise in mental health ethics and law.

**Methods:**

Seventeen semi-structured interviews with users, family members and professionals, and one focus group with five researchers were conducted. The data was analyzed using qualitative content analysis.

**Results:**

Six opportunities and five risks of SBDs were identified. The opportunities were promotion of autonomy and self-efficacy of users, relief of responsibility for family members, early intervention, reduction of (perceived) coercion, positive impact on the therapeutic relationship and enhancement of professionals' confidence in decision-making. The risks were problems in the assessment of mental capacity, inaccurate information or misinterpretation, increase of coercion through misuse, negative impact on the therapeutic relationship due to noncompliance with SBDs, and restricted therapeutic flexibility and less reflection on medical decision-making. Stakeholders tended to think that the opportunities of SBDs outweigh their risks, provided that appropriate control and monitoring mechanisms are in place, support is provided during the drafting process and the respective mental healthcare setting is sufficiently prepared to implement SBDs in practice.

**Conclusions:**

The fact that stakeholders consider SBDs as an opportunity to improve personalized crisis care for people with bipolar disorder indicates that a debate about the legal and clinical implementation of SBDs in Germany and beyond is necessary.

## Introduction

Self-binding directives (SBDs), also known as Ulysses contracts or Ulysses arrangements, are a special type of psychiatric advance directive by means of which mental health service users (henceforth: users) can consent in advance to involuntary hospital admission and involuntary treatment during future mental health crises (1). Self-binding directives and joint crisis plans differ from standard psychiatric advance directives in that the latter type of instrument can be drafted and signed by the user alone, whereas the former types of instruments are essentially collaborative and are signed by the user and a representative of the treatment team. Self-binding directives differ from both standard psychiatric advance directives and joint crisis plans in two ways: firstly, they enable users to agree in advance to involuntary hospital admission or treatment in the event of a future mental health crisis, and secondly, they cannot be revoked under the circumstances for which they were written (2–5). Treatment includes not only medication but also de-escalation strategies to prevent further escalation in a crisis situation. Explicit legal provisions for SBDs exist in the Netherlands (1). Advance directives in Germany are legally regulated and binding in both somatic and mental healthcare (6, 7). However, there are currently no explicit legal provisions for SBDs in German law.

Self-binding directives can be particularly suitable for users with mental disorders characterized by “fluctuating capacity,” such as bipolar and psychotic disorders (4). Fluctuating capacity denotes the alternation of phases in which people have mental capacity with phases in which they lack mental capacity. Based on previous experiences of mental health crises, users can use SBDs to plan their treatment during future mental health crises in advance and stay in control of their lives and treatment (8–10). In our study, we focus on users with bipolar disorder, since this mental disorder represents a paradigmatic case of fluctuating capacity. Especially during manic episodes, users may lack mental capacity and pose a risk of harm to self or others, as a result of which involuntary hospital admission and treatment may be necessary to prevent substantial harm.

Researchers from psychiatry, law, medical ethics and philosophy have described several ethical opportunities and risks of SBDs from a theoretical point of view. They referred to an enhancement of the users' autonomy and well-being, the improvement of the therapeutic relationship and relationships with family members, the possibility of early interventions and a relief for substitute decision-makers as benefits of SBDs (3, 4, 11–15). On the other hand, they discussed ethical risks of SBDs related to self-paternalism, increased susceptibility to undue influence, an increased use of coercion, the impossibility of changing one's own opinion and expired consent (16–19).

Up to now, there has been little empirical knowledge available about the attitudes of stakeholders, notably people with bipolar disorder, toward SBDs. It is known, however, that people with bipolar disorder have a high interest in advance decision-making in general (9, 10) and that many people with bipolar disorder in the UK endorse the idea of SBDs (20, 21). Furthermore, qualitative studies from the Netherlands showed that various stakeholders, including users, family members and mental health professionals, see both opportunities and risks of SBDs. The opportunities include timely intervention and avoiding harm, and the risks include undue influence during the drafting process and premature hospital admission (22, 23). To the best of our knowledge, no empirical studies on SBDs in the context of German mental healthcare have been conducted thus far.

The aim of our study was to investigate whether stakeholders share the primarily theoretical views of medical ethicists and legal or other scholars on SBDs and which additional aspects they might identify from a practical or personal perspective. Our research question was: What opportunities and risks of SBDs do stakeholders and researchers with expertise in mental health ethics and law (henceforth: researchers) in Germany see?

## Methods

Our methodological approach was qualitative content analysis according to Kuckartz (24). This enabled us to assess whether the opportunities and risks of SBDs referred to in the theoretical literature are shared by the stakeholders and researchers in Germany, and add novel and unexplored aspects to the literature inductively. We obtained ethical approval for this study from the Research Ethics Committee of the Medical Faculty of the Ruhr University Bochum, Germany, registration no. 19-6809. All participants were informed both orally and in writing and gave their written informed consent prior to their participation.

### Sampling and data collection

It is important in debates on ethically controversial interventions with a potentially high impact on users and related stakeholders to consider the perspectives of the people who would be most affected by changes in the legal system or clinical practice. For these reasons, we included three stakeholder groups in our study: users, family members of people with bipolar disorder (henceforth: family members) and professionals working with people with bipolar disorder (henceforth: professionals). Specifically, these were six people with a self-reported diagnosis of bipolar disorder and experience with inpatient treatment (on either a voluntary or involuntary basis), six family members (including parents and spouses) and five professionals (a psychiatrist, a psychologist, a social worker, an advanced practice nurse and a legal guardian). Furthermore, because the ethico-legal context is relevant for the evaluation of SBDs, especially regarding a possible implementation of SBDs in the German mental healthcare system, we included five researchers with expertise in this area in our study.

Several of the participants had personal experiences both as a user and family member, user and professional or professional and researcher. A total of 22 participants took part in the study. They varied by gender (14 women and 8 men) and age (from 25 to 80 years). The participants were approached via email, telephone or personally through various self-help groups of users and family members and through academic or clinical working groups and institutions relevant to the topic.

The data were collected in Germany between February 2020 and March 2021. We started the study with a focus group of the five researchers but adapted our method for data collection in response to the COVID-19 pandemic and the associated restrictions. We continued the data collection with semi-structured qualitative interviews with the other 17 participants via telephone (eight), video call (three) and face-to-face (six). The data collection was concluded when theoretical saturation was reached. Interviews lasted between 25 and 60 min. The face-to-face interviews took place at the interviewee's workplace or home, or in a mental health hospital. The focus group lasted 77 min and was conducted in person at a university facility. The focus group was conducted by MS and JG and the interviews by SP, MF and JG. The focus group and interviews were audio-recorded and transcribed verbatim. We then pseudonymized the transcripts and the interview excerpts cited were translated into English after analysis with the help of a native speaker. Punctuation was added on some occasions to improve readability.

The focus group and the interviews were semi-structured. The topic guide included questions about personal experiences with advance directives, opportunities and risks of SBDs, preferred criteria for SBD completion, application and revocation, and views on the need for monitoring mechanisms to reduce risks. The topic guide was developed according to the content analysis approach based on categories that we had compiled from the literature. Besides the study information sheet and the informed consent form, all participants of the focus group and the interviews received an information sheet about SBDs. In addition, the interviewers briefly explained the concept of an SBD and its differences to other types of psychiatric advance directives to the study participants at the beginning of the focus group and the interviews, respectively.

### Data analysis

We followed the principles of qualitative content analysis according to Kuckartz (24) for the analysis. As a first step, the research team jointly developed a category guide deductively based on the existing literature on SBDs. The qualitative content analysis according to Kuckartz allows for a deductive and inductive procedure of data analysis, and this enabled us to compare the theoretical views of academics on SBDs with the views of stakeholders. The authors SP and MF used the category guide to code all transcripts alone, compared the codes and discussed any discrepancies. During the coding process, additional categories were developed inductively, and some existing categories were changed according to the data. We used the software MAXQDA 2020 Standard (VERBI Software GmbH, Berlin, Germany). The authors MS, AG, JV, and JG were included in the analysis in team meetings to ensure the quality criterion of intersubjective traceability through the perspectives of different disciplines. Our research team included researchers with backgrounds in sociology, medical ethics, medicine, clinical psychiatry and philosophy.

## Results

We present the results of the analysis from a category-oriented perspective in what follows. A case-oriented presentation according to the four groups (users, family members, professionals and researchers) does not seem appropriate to us, since the analysis does not show clearly assignable differences between these groups.

### Opportunities of SBDs

Table 1 provides an overview of the opportunities of SBDs from the study participants' point of view.

**Table 1:** Opportunities of SBDs.

1. Promotion of autonomy and self-efficacy of users
2. Relief of responsibility for family members
3. Early intervention
4. Reduction of (perceived) coercion
5. Positive impact on the therapeutic relationship
6. Enhancement of professionals' confidence in decision-making

#### Promotion of autonomy and self-efficacy of users

Participants emphasized that SBDs provide an opportunity to strengthen the autonomy and self-efficacy of users. This can result from the necessary reflection on one's own mental disorder during the drafting process, as one user describes:

*More active involvement of the user, because it is also a clear call to those who are ill to concern themselves more with the situation. I think it raises awareness. [...] For me, it would perhaps be the concreteness [of the information in the SBD][…], it would simply be a step toward certainty. [...] I think it gives me more certainty that my social network gets in touch with me, then the next step is clearer for me, and also for my social network. (User 5)*

The quote also shows that the self-determined planning of future crisis situations is important to strengthen transparency and certainty for users and for their relatives, who should be enabled to act in accordance with the users' preferences in mental health crisis situations.

#### Relief of responsibility for family members

Increased autonomy of users through the development of preferences for a crisis situation in advance is, at the same time, a form of relief of responsibility for their family members. It was pointed out in the focus group that family members no longer have to make substitute decisions, which relieves them of responsibility and promotes users' autonomy. This was also referred to in the following quote from a family member:

*Personally, I would support this. [...] Simply because I know that family members are relatively helpless here and I am grateful for all support that family members can have here. Of course, in connection with the consent of the persons concerned, if possible. But that you are not constantly left out in the cold because you don't know what you can do and how things can continue. That is why I would be pleased if such a Ulysses arrangement officially saw the light of day in the foreseeable future. (Family member 1)*

#### Early intervention

The possibility of early intervention emerged from the data as a major theme among stakeholders. Early intervention can include community support services, assertive community treatment, admission to a mental health hospital or provision of medication. An advantage of SBDs identified by stakeholders is that involuntary admission and treatment can be already arranged before the high legal thresholds defined in German guardianship law or the mental health laws of the German states have been reached. Stakeholders thought that earlier admission based on an SBD need not proceed involuntarily but can also proceed voluntarily if SBDs are helpful in reminding users of their considered treatment preferences set down in a step-by-step plan. Overall, users and family members viewed earlier admission, including involuntary admission, as a possibility for help and treatment rather than as coercion. By contrast, early intervention by means of involuntary medication was evaluated more critically.

Stakeholders regarded early hospital admission as helpful because of the opportunity to protect users against various forms of harm. This includes protection from feelings of shame and guilt, damage to health, financial damage and damage to social relationships. Users particularly pointed out the opportunity of using an SBD to protect themselves from feelings of shame and guilt. They hoped that early hospital admission would keep them from doing things for which they would feel ashamed and guilty afterwards.

*When I was really manic at that time and colleagues tried to arrange a legal guardian for me so that I could be committed, and that didn't work out and I really destroyed a lot of things. I often wished that it would have worked out back then, that it would have been enough to prevent these consequences, which then also led to the fact that the manic who wakes up has to fight with feelings of guilt. (User 3)*

A second important consequence of early hospital admission was the possibility of the reduction of the duration of hospitalization, which one user emphasized in the following quote:

*The earlier you are admitted, the faster you will usually improve again, so that you can be discharged earlier. Based on my own experience, I think that it would be really important if it could take place earlier […] simply to prevent grave, rapid deterioration earlier, which would otherwise become dangerous. (User 6)*

#### Reduction of (perceived) coercion

Participants evaluated SBDs positively regardless of their attitude toward coercion in mental healthcare. They believed that SBDs cannot prevent all forms of coercion. Instead, they tended to think that SBDs would allow for the application of coercion at an earlier stage in a mental health crisis, which might lead to a quicker remission of symptoms and, thus, reduce the total amount of coercion used during an inpatient treatment. Early involuntary hospital admission based on an SBD, for instance, could prevent involuntary medication, because a therapeutic and supportive environment in a hospital could help users to manage their crisis at an earlier stage. However, it is important that the respective mental healthcare environment has supportive resources in the form of sufficient and well-trained staff. Coercion can also be avoided when SBDs are used to remind users of their considered step-by-step treatment preferences and convince them to accept hospital admission voluntarily.

*This is essential, not to regard a hospital as a place of terror but as a place of help, that would have the consequence, and I have heard this from clinics, from clinicians who offer this [joint crisis plans], that here, a patient who feels, “Oh, now it's getting critical,” voluntarily admits himself for a few days and, thus, intercepts a crisis. Because he has the confidence that, “Yes, the clinic will pay attention to my wishes. The clinic knows what medication I definitely don't want, how they have to deal with me at the beginning of mania and so on.” (Family member 5)*

According to the quote, an important prerequisite for consenting to early admission is that users can trust that their joint crisis plans or SBDs will be recognized by professionals.

Furthermore, users and professionals pointed out that coercion might be experienced less negatively in retrospect if its use is based on an SBD and, hence, on the precedent autonomy of the user.

*Interviewee: Well, I wanted it [compulsory treatment] that way then. I determined it beforehand in my clear head that I would be treated compulsorily. […]. Ulysses who lets himself be tied down. […], where I then say to myself, I want this network [the procedure in a crisis agreed in an SBD] to take control now*.

*Interviewer: Do you think that would make a difference in the perception, or later, in retrospect, of the compulsory treatment?*

*Interviewee: For sure. I wanted it. I wanted to be tied up, so to speak, and to be injected with the medication. (User 4)*

#### Positive impact on the therapeutic relationship

Study participants stated that SBDs could have a positive impact on the therapeutic relationship because drafting an SBD requires regular exchanges between users, professionals and family members in non-acute phases of the mental disorder. Professionals can use SBDs at the beginning and during a mental health crisis to remind users of jointly made treatment agreements. The SBD can be an expression of a successful therapeutic relationship, as the following quote from a professional shows:

*I rather imagine that the people who get involved in this Ulysses arrangement in the first place can experience it as perhaps a kind of empowerment: “I can have a say in how I'm treated next time, if I, if something happens to me, if I'm no longer well.” I think involving patients in treatment is always good. […] that's always good for the relationship between the professional and the patient; it's always good when patients have supported the decision, at least at a certain point, and have helped to decide. (Professional 2)*

In order that SBDs have the effect of improving the therapeutic relationship, it is important that professionals comply with SBDs during crises so that users can be confident that professionals will adhere to their SBDs.

#### Enhancement of professionals' confidence in decision-making

Exchanges between users and professionals during the SBD drafting process can offer increased clarity for professionals regarding users' treatment preferences during mental health crises. It would enhance professionals' confidence in decision-making especially in crisis situations in which the legally defined criteria for involuntary hospital admission are not yet fulfilled, but admission is, nevertheless, requested by users in their SBD. As SBDs create transparency about the preferences of users, they give professionals the opportunity to evaluate situations correctly and act accordingly, which may have a de-escalating effect and, thus, help to prevent coercion. One user explained how the behavior of others, including professionals, can contribute to an escalation or de-escalation of a crisis:

*A crisis is never just one event. It's a whole strand that can sometimes last for two or three weeks. They are encounters that come to a head. That's how I would reflect on crisis. Everyone always says, “Now the crisis is here.” No. It's not like that. The crisis builds up and consists of encounters. Either of helpful encounters or of aggravating encounters. And the people in the encounters, if they have more information, then they can become helpers instead of escalating the situation. (User 2)*

### Risks of SBDs

Table 2 provides an overview of the risks of SBDs from the study participants' point of view.

**Table 2:** Risks of SBDs.

1. Problems in the assessment of mental capacity
2. Inaccurate information or misinterpretation
3. Increase of coercion through misuse
4. Negative impact on the therapeutic relationship due to noncompliance with SBDs
5. Restricted therapeutic flexibility and less reflection on medical decision-making

#### Problems in the assessment of mental capacity

The stakeholders emphasized both the difficulty of determining the lack of mental capacity in the situation when an SBD should be applied and the difficulty of assessing the presence of mental capacity for SBD revocation after a crisis. Both situations involve the risk of misjudgement: mental capacity can be either unjustifiably denied or unjustifiably granted. To address this risk, stakeholders proposed an assessment of mental capacity by more than one psychiatrist, preferably the treating psychiatrist and an independent psychiatrist.

*There is always the possibility that the situation is misjudged by the doctor. I would want several doctors, not one doctor alone, to assess the applicability of the directive, that the capacity to consent is no longer given. That it is not one doctor alone who decides. (User 6)*

By contrast, the stakeholders expressed fewer concerns about the determination of mental capacity at the time of SBD creation, since at this time, users are generally not in a mental health crisis.

#### Inaccurate information and misinterpretation

Participants tended to see a risk of unclear or inaccurate information in SBDs and a related risk of misinterpretation of SBD instructions. Stakeholders rated the risk of unclear or inaccurate information as low, provided that users have received support during the drafting process. Stakeholders suggested the development of SBD templates and the involvement of various people in the drafting process as suitable support interventions. Depending on the user's preferences, these support people could be close relatives or friends, treating psychiatrists or psychotherapists, or social workers. It was considered essential that support people be well acquainted with users and have regular contact with them.

*That [inaccurate or unclear information in the SBD] will also be a problem. But you can prevent that by making the directive with a doctor or a psychologist. I think it's problematic to fill out such an advance directive by yourself, alone. […] it's best to discuss the whole thing with a doctor, a psychiatrist or a psychologist. (Family member 3)*

Researchers saw a risk of misinterpretation of SBDs in the detection of early warning signs by family members and professionals. Stakeholders, however, did not express this concern. They stated that relatives and treating professionals are usually familiar with the early warning signs of the respective user.

#### Increase of coercion through misuse

All stakeholders expressed the concern that lowering the threshold for coercion by means of self-determined conditions for intervention in SBDs could lead to an increased use of coercion. As an important protective measure, users mentioned a precise arrangement about the content of SBDs and having a choice about which professional is involved in the drafting process.

*If you set a threshold lower, the risk of abuse is there, of course. That is always the case. […] in relation to the illness, I'm now quite clear that I trust professionals first and foremost. I would select them [professionals] in consultation with the doctors and, therefore, my mistrust would not be so high. (User 5)*

Professionals and researchers raised the concern that even with precise information in an SBD, the latter could be misused and lead to an increased use of coercion. Misuse could result from family conflicts, as one professional pointed out in the following quote:

*We also have many family members here who have problems with their family member, that is, the patient. On a relationship level, on all possible levels. And somehow the perception of illness is mixed up with simple communication problems, with other ideas about how life should be organized, and so on. And no one can really separate them. And in this situation of diversity of interests, such a family member pulls out the SBD and says: “But now the time has come.” (Professional 3)*

The professional here implies that relatives might have too much power to decide when the SBD comes into force, which could be problematic in the case of personal conflicts or conflicting interests.

#### Negative impact on the therapeutic relationship due to noncompliance with SBDs

Noncompliance with SBDs by clinicians can have a negative impact on the therapeutic relationship. Researchers and stakeholders mentioned the risk that users would perceive noncompliance as a breach of trust in relation to professionals and confidence in the mental healthcare system.

*Participant 5: That the contents of an SBD by the hospital, which is then supposed to implement it, are not implemented in such a way, that is, the measures are not implemented as they have just been determined by the patient, for whatever reasons. Or that there are contradictions in there. That it was formulated somehow misleadingly in the SBD*.

*Participant 3: With the result of a loss of trust. (Focus group with researchers)*

#### Restricted therapeutic flexibility and less reflection on medical decision-making

Professionals and researchers saw a risk of restricted therapeutic flexibility due to treatment instructions in an SBD. They raised the concern that professionals could be bound by SBD instructions and would no longer have the opportunity to choose the best treatment option available. Restricted therapeutic flexibility was also seen as having the effect that professionals might be inclined to simply act according to SBD instructions and no longer reflect critically on their medical decisions. The latter risk was discussed primarily among researchers:

*Participant 3: But it can also simply be that if it [content of SBD] is fixed so concretely in writing and you stand there as a practitioner and say, “Great, that's how it was last time, but this time everything is different,” for whatever reason. Liver values are not correct or something else comes up that no one has considered, and then you […] are somehow bound to this directive, so it really becomes legally difficult in the end. [...] One could also understand it as a carte blanche for the practitioners, without them really thinking about the medical indication again in whatever sense. But say, “Oh there it is, then we give it first.”*

*Moderator 1: Any other risks?*

*Participant 5: Perhaps that completely different alternatives disappear from view, non-pharmacological ones I mean. (Focus group with researchers)*

## Discussion

The stakeholders and researchers participating in our study identified various opportunities and risks of SBDs in the context of German mental healthcare. Their views support, challenge and complement the opportunities and risks identified in the theoretical debate on SBDs. They confirm opportunities, such as the promotion of autonomy, the relief for family members as substitute decision-makers, the possibility of early intervention, the reduction of the duration of involuntary hospitalization, the improvement of therapeutic relationship and the enhancement of professionals' confidence in decision-making (3, 4, 11–15). Stakeholders did not raise any concerns about self-paternalism, the impossibility of changing one's own mind and expired consent (16–19). This could indicate that these theoretical concerns are not that relevant in clinical practice. Our findings complement the risks of SBDs discussed in the theoretical literature. Additional risks identified in our study include a negative impact on the therapeutic relationship due to noncompliance with the SBD, inaccurate information or misinterpretation, as well as restricted therapeutic flexibility and less reflection on medical decision-making. These risks should be considered in both future theoretical analyses and practical initiatives to implement SBDs in mental healthcare.

Many opportunities and risks of SBDs identified by stakeholders and researchers in our study seem to also apply to psychiatric advance directives and joint crisis plans. A benefit uniquely pertaining to SBDs is enabling early intervention in the form of involuntary admission and treatment. A risk pertaining uniquely to SBDs is an increase of coercion through misuse of the instrument. It is unlikely that the partial overlap of opportunities and risks is explained by a lack of knowledge among study participants about the differences between SBDs, on the one hand, and psychiatric advance directives, on the other, because study participants were explicitly informed about these differences through a brief educational intervention. Instead, the overlap in perceived opportunities and risks should not be surprising given that an SBD is a specific type of psychiatric advance directive.

Overall, stakeholders included in our study tended to see more opportunities than risks attached to SBDs. However, participants stressed that a good and sufficiently prepared mental healthcare system represents a prerequisite for these opportunities of SDBs to become effective in practice. Such a mental healthcare system is characterized in the view of participants by staff that includes peer support workers, is sufficiently available, and which is well-trained in de-escalation strategies and the implementation of SBDs. Accordingly, the mere introduction of SBDs into a mental healthcare setting that is not sufficiently prepared for its implementation is not likely to change much for the better.

Participants also tended to think that the risks attached to SBDs can be addressed by support interventions and control mechanisms. The support interventions proposed included the provision of an SBD template ensuring that all necessary information is included in the document and the joint creation of an SBD by users, professionals and trusted people, which can ensure that SBD instructions are feasible and compatible with practice standards and are known by all responsible parties in the event of a crisis. The control mechanisms proposed included the assessment of mental capacity by the treating psychiatrist and an independent psychiatrist, and the assessment of early warning signs described in the SBD by two professionals and someone close to the user.

Overall, our findings suggest that SBDs can only yield their benefits based on a relationship of mutual trust between users and professionals. These findings are supported by results from other empirical studies. Gergel et al. (21) reported for the UK context that most respondents with a self-reported diagnosis of bipolar disorder endorsed SBDs. In the Netherlands, Gremmen et al. (23) found that the users' confidence in future mental health treatment is strengthened by reflection on their mental disorder during the SBD drafting process. Transparency regarding treatment in future crisis situations can also have a positive effect on promoting autonomy, self-confidence and self-efficacy of users. The study by Varekamp (22), also conducted in the Netherlands, equally showed that both users and psychiatrists consider promoting autonomy, enabling timely intervention and avoiding harm as the most important opportunities of SBDs. In contrast to our study, concerns about the determination of a lack of mental capacity in relation to the application of SBDs were not reported in the Dutch studies. Conversely, a concern about the scarcity of available hospital beds reported in the study by Varekamp (22) was not a prominent theme in our data. An explanation of this latter difference could lie in the beds per capita of the two countries. In 2020, the Netherlands had 0.79 psychiatric beds per 1000 inhabitants, compared to 1.30 in Germany (25).

Our study provides a further specification of the notion of harm. We found that protection from harm can mean protection from feelings of shame and guilt, damage to health, financial damage and damage to social relationships. Interestingly, users did not tend to see early intervention in mental health crises based on an SBD, even when involuntary, as a form of coercion but rather as a form of help and treatment. The latter includes compliance with a step-by-step treatment plan set out in the SBD with the aim of preventing escalation of an emerging mental health crisis situation.

## Limitations

A limitation of our study is that there are no explicit legal provisions for SBDs in Germany to date and that, consequently, participants' statements were based on hypothetical considerations rather than their own practical experience with SBDs. This limitation is mitigated, however, by the fact that most participants had prior practical experience with either psychiatric advance directives or joint crisis plans, both of which are legally binding in Germany under specific conditions, and were educated briefly about the nature and specificities of SBDs before the interview or focus group.

Another limitation of our study is the potentially limited heterogeneity of the sample due to selection bias. We assume that participants with an interest in advance decision-making were more likely to agree to participate in the study. However, our results do not show a homogeneous positive assessment of advance decision-making but include critical statements as well. Furthermore, the heterogeneity of the sample is limited by the fact that we focused on people with bipolar disorder. It seems plausible, however, that our research findings also hold for other types of disorders involving fluctuating capacity, such as psychotic disorders or severe depression. Future research should also include people with these types of mental disorders.

## Conclusions

The fact that stakeholders consider SBDs as an opportunity to improve personalized crisis care for people with bipolar disorder indicates that a debate about the legal and clinical implementation of SBDs in Germany and beyond is necessary. Strategies to address potential risks and realize the opportunities of SBDs in clinical practice should be a topic in this debate. Although the focus of our study was on bipolar disorder, it is plausible to assume that SBDs can also be helpful for people with other types of mental disorders involving fluctuating capacity. Further quantitative research is required to assess whether SBDs receive support in a representative sample of stakeholders and which forms of support and monitoring for SBDs are endorsed most widely.

## Data availability statement

The data cannot be shared publicly as this may compromise the privacy of research participants. The anonymized data can be made available by the corresponding author upon reasonable request.

## Ethics statement

This study was reviewed and approved by the Research Ethics Committee of the Medical Faculty of the Ruhr University Bochum, Germany, registration no. 19-6809. All study participants were informed both orally and in writing and provided their written informed consent prior to their participation.

## Author contributions

JG and MS made the initial design of the study and all authors contributed to working out the design in detail. MF developed the topic guide based on input from AG and MS, and all authors provided feedback on it. MS and JG carried out the focus group and SP, MF, and JG the interviews. SP and MF carried out the data analysis. Findings were discussed continuously with JG and MS and in team sessions with the other authors. SP and MF jointly wrote the methods and results section, and SP, JG, and MS jointly wrote the background and the discussion section. All authors contributed to the article and approved the submitted version.

## Funding

This research is part of the project SALUS (2018–2024) and supported by a grant from the German Federal Ministry of Education and Research (no. 01GP1792).

## Conflict of interest

The authors declare that the research was conducted in the absence of any commercial or financial relationships that could be construed as a potential conflict of interest.

## Publisher's note

All claims expressed in this article are solely those of the authors and do not necessarily represent those of their affiliated organizations, or those of the publisher, the editors and the reviewers. Any product that may be evaluated in this article, or claim that may be made by its manufacturer, is not guaranteed or endorsed by the publisher.
